# Tau pathology in Creutzfeldt‐Jakob disease revisited

**DOI:** 10.1111/bpa.12411

**Published:** 2016-08-02

**Authors:** Gabor G. Kovacs, Jasmin Rahimi, Thomas Ströbel, Mirjam I. Lutz, Günther Regelsberger, Nathalie Streichenberger, Armand Perret‐Liaudet, Romana Höftberger, Pawel P. Liberski, Herbert Budka, Beata Sikorska

**Affiliations:** ^1^ Institute of Neurology, Medical University of Vienna, and Austrian Reference Center for Human Prion Diseases Vienna Austria; ^2^ Prion Disease Laboratory, Pathology and Biochemistry Groupement Hospitalier Est, Hospices Civils de Lyon/Claude Bernard University Lyon France; ^3^ Institut NeuroMyogène CNRS UMR 5310 ‐ INSERM U1217 Lyon France; ^4^ Centre de Recherche en Neurosciences de Lyon (Laboratoire BioRaN) Université Claude Bernard Lyon 1 – CNRS UMR5292 – INSERM U1028 Lyon France; ^5^ Department of Molecular Pathology and Neuropathology Medical University of Lodz Lodz Poland; ^6^ Institute of Neuropathology, University Hospital Zurich Zurich Switzerland

**Keywords:** ARTAG, cerebrospinal fluid, Creutzfeldt‐Jakob disease, PART, prion, tau

## Abstract

Creutzfeldt‐Jakob disease (CJD) is a human prion disease with different etiologies. To determine the spectrum of tau pathologies in CJD, we assessed phospho‐Tau (pTau) immunoreactivities in 75 sporadic CJD cases including an evaluation of the entorhinal cortex and six hippocampal subregions. Twelve cases (16%) showed only small tau‐immunoreactive neuritic profiles. Fifty‐two (69.3%) showed additional tau pathology in the medial temporal lobe compatible with primary age related tauopathy (PART). In 22/52 cases the lower pTau immunoreactivity load in the entorhinal cortex as compared to subiculum, dentate gyrus or CA4 region of the hippocampus was significantly different from the typical distribution of the Braak staging. A further 11 cases (14.7%) showed widespread tau pathologies compatible with features of primary tauopathies or the gray matter type of ageing‐related tau astrogliopathy (ARTAG). Prominent gray matter ARTAG was also observed in two out of three additionally examined V203I genetic CJD cases. Analysis of cerebrospinal fluid revealed prominent increase of total tau protein in cases with widespread tau pathology, while pTau (T181) level was increased only in four. This correlated with immunohistochemical observations showing less pathology with anti‐pTau T181 antibody when compared to anti‐pTau S202/T205, T212/S214 and T231. The frequency of tau pathologies is not unusually high in sporadic CJD and does not precisely relate to PrP deposition. However, the pattern of hippocampal tau pathology often deviates from the stages of Braak. Currently applied examination of cerebrospinal fluid pTau (T181) level does not reliably reflect primary tauopathies, PART and ARTAG seen in CJD brains.

## Introduction

Prion diseases are rapidly progressive lethal neurodegenerative disorders with various etiologies, including sporadic (idiopathic), genetic and acquired forms 37. The most frequent is sporadic Creutzfeldt‐Jakob disease (sCJD). Prion diseases are major models for other neurodegenerative conditions because a pathological conformer of the physiological prion protein (PrP) is able to transmit the disease, hence the term transmissible spongiform encephalopathies. Disease‐associated PrP deposits mostly in the form of synaptic, perivacuolar, plaque‐like or amyloid plaque type deposits 37. Immunoreactivity for PrP shows excellent correlation with molecular subtypes defined based on the Western blot pattern of the protease‐resistant PrP and the polymorphic codon 129 (Methionine/Valine, MV) of the prion protein gene (*PRNP*) 53, 54. Genetic prion disease either shows the morphology of CJD or is characterized by multicentric amyloid plaques as in Gerstmann‐Sträussler‐Scheinker disease (GSS) or thalamic degeneration such as fatal familial insomnia (FFI) 37.

Neurodegenerative diseases are classified according to the clinical symptoms, anatomical regions affected and proteins involved in the pathogenesis 35. While immunoreactivity for amyloid‐β (Aβ) or PrP is located predominantly extracellularly, major proteins that deposit intracellularly include tau, α‐synuclein, TAR DNA binding protein 43 (TDP‐43) or fused in sarcoma protein (FUS) 35. A considerable number of diseases, summarized as tauopathies, show tau pathology 34. Tau is a microtubule‐associated protein encoded by a single gene (*MAPT*). Tauopathies are classified based on the presence of isoforms of tau, which associate with different patterns of insoluble tau on Western blotting. Three isoforms with 0, 1 or 2 inserts contain three microtubule‐binding repeats (R) and are designated as 3R tau; and three isoforms, also with 0, 1 or 2 inserts, containing four microtubule‐binding repeats, are designated as 4R tau 34. While Alzheimer disease (AD) and neurofibrillary tangle (NFT) predominant dementia features both 3R and 4R isoforms, corticobasal degeneration (CBD), progressive supranuclear palsy (PSP), globular glial tauopathies (GGT) and argyrophilic grain disease (AGD) are thought to be 4R predominant, in contrast to Pick's disease, which is a 3R isoform predominant tauopathy 34. Several diseases may associate with tau pathologies, however, two conditions described predominantly in the ageing brain need to be emphasized. One is primary age‐related tauopathy (PART), which designates cases with NFTs in the medial temporal lobe without significant concomitant Aβ depositions 8. The other is ageing‐related tau astrogliopathy (ARTAG), which specifies astrocytic tau immunoreactivities such as thorn‐shaped astrocytes (TSA) or granular–fuzzy astrocytes (GFA) detectable in various anatomical regions in subpial, subependymal, perivascular and gray or white matter locations 40.

In most of the prion disease forms small tau immunoreactive neuritic profiles are seen 39, 59. Furthermore, NFT pathology is considerable in GSS and PrP cerebral amyloid angiopathy 14, 15, 16, 17, 18, 19, 20, while some forms of genetic CJD 46 and even FFI 30 and variably protease sensitive prionopathy 12, 72 cases may show neuronal tau pathologies. During our surveillance of prion diseases in several cases we noticed that the hippocampal distribution of neuronal tau pathology shows deviation from the classical stages of NFT pathology as described by Braak and colleagues. Furthermore, we observed prominent astroglial tau pathology in several cases. This prompted us to perform a meticulous analysis of the hippocampi of sCJD cases and to screen several anatomical regions for phospho‐Tau (pTau) pathology.

## Material and Methods

### Case selection

We included 75 consecutive sCJD cases from the collection of the Austrian Reference Centre for Human Prion diseases, where all areas for the full state‐of‐the‐art characterization of different neurodegenerative disorders were available 38. For comparison of the complex tauopathy seen in a subset of sCJD cases, we included three cases (two from France and one from Austria) with the V203I mutation of the *PRNP* gene. Genetic analysis was performed using genomic DNA isolated from blood samples or frozen brain tissue as published before in the frame of CJD surveillance 46. Human biological samples and associated data were obtained from the CJD Surveillance and KIN‐Biobank Medical University of Vienna (Ethics Committee approval: 396/2011) and the Cardiobiotec Biobank (CRB‐HCL Hospices Civils de Lyon BB‐0033‐00046). All tissue samples were obtained according to Austrian and French legislations.

### Neuropathology

For the detailed study of tau pathologies we used formalin‐fixed, paraffin‐embedded blocks. Sections of cortical areas, basal ganglia, thalamus, hippocampus, amygdala, midbrain, pons, medulla oblongata and cerebellum with dentate nucleus were examined. For all cases we used anti‐tau AT8 (pS202 + pT205, 1:200), and in cases with complex tauopathies we applied further antibodies including AT100 (pT212 + pS214, 1:200), AT180 (pT231, 1:200) and AT270 (pT181, 1:200), all from Thermo Fisher Scientific, Rockford, IL, USA; furthermore, anti‐4R tau (RD4, 1:200, Upstate, Charlottesville, VA, USA) and anti‐3R tau (RD3, 1:2000, Upstate). Gallyas staining was used in selected cases. For fluorescence double labeling the following antibodies were used: anti‐glial fibrillar acidic protein (GFAP, rabbit, 1:3000, Dako), AT8, and anti‐PrP (1:2000, 12F10, Cayman Chemical, Ann Arbor, MI, USA). The fluorescence‐labeled secondary antibodies were Alexa Fluor (AF) 555 donkey anti‐mouse IgG (1:200, Molecular Probes, Inc., Eugene, OR, USA) and AF 488 goat anti‐rabbit (1:200, Molecular Probes, Inc.). The following combinations were applied: polyclonal AT8 (AF 555)/GFAP (AF488) and 12F10 (AF555)/GFAP (AF488). We evaluated double immunofluorescent labeling with a Zeiss LSM 510 confocal laser microscope.

### Semiquantitative evaluation of hippocampal tau pathology

Phospho‐tau immunoreactivity (AT8) was evaluated semiquantitatively (0‐no immunoreactivity or tiny dots characteristic of CJD; 1‐mild; 2‐moderate; 3‐prominent immunoreactivity) in seven subregions (dentate gyrus, the CA1‐4 regions of the hippocampus, the subiculum and the entorhinal cortex). We scored the neuronal (cell body) and neuritic (threads) tau deposits separately.

### Examination of tau and phospho‐tau in the cerebrospinal fluid

Concentrations of total tau protein and phospho‐tau were determined in cerebrospinal fluid by ELISA using INNOTEST^®^ hTAU Ag and INNOTEST^®^ PHOSPHO‐TAU_(T181P)_ assays (Fujirebio Europe N.V., Gent, Belgium) following the recommendations of the supplier.

### Statistical analysis

Chi^2^ test was used to compare the proportion of tau pathologies in different molecular subtypes of CJD. Kruskall–Wallis (K‐W) and Mann–Whitney (M‐W) tests were used to compare age, duration of illness and the scores of neuritic and neuronal tau immunoreactivities in different subregions and different case groups. We correlated the scores with age and duration of illness using the Spearman's rank correlation coefficient rho.

## Results

### Overview and stratification of cases

The mean age at death (± standard error) of sCJD cases was 71.2 ± 0.7 years (range 56–88; 36 men). The following molecular subtypes 53, 54 were represented: MM/MV‐1 (47 cases); MM‐2C (5 cases); MV‐2K (6 cases); VV‐1 (2 cases) and VV‐2 (15 cases).

**Figure 1:**
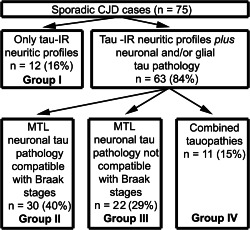
Stratification of tau pathologies in the studied sporadic Creutzfeldt‐Jakob disease cases.

**Figure 2:**
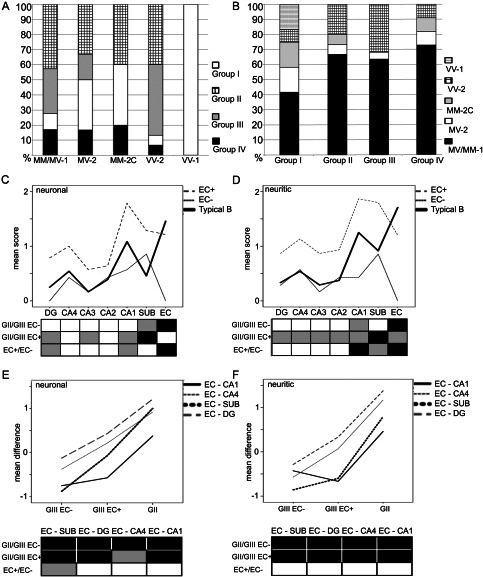
Distribution of tau pathology groups (I–IV) in molecular subtypes of sporadic Creutzfledt‐Jakob disease **(A)** and of different molecular subtypes in each group of tau pathology **(B)**. Graphic representation of mean scores of neuronal **(C)** and neuritic **(D)** tau immunoreactivity in cases with typical Braak (“B”) distribution (group II, GII) and those with atypical distribution (group III, GIII) including entorhinal cortex (EC) positive (+) and negative (−) subtypes. Graphic representation of the subtraction of mean scores of neuronal (E) and neuritic (F) tau immunoreactivity in the EC and different subregions in cases of group II and those with group III (EC− and EC+) subtypes. Boxes below the graphics in C–F indicate significance levels in Mann–Whitney tests (white: *P* > 0.05, gray: *P* < 0.05, black: *P* < 0.01). DG: dentate gyrus; CA: cornu ammonis; SUB: subiculum.

Based on the distribution of tau pathology, four groups were distinguished (summarized in Figure 1). In group I (*n* = 12; 16%; 6 men; age: 65.0 ± 1.4) only tiny (<3 μm) dots of AT8 immunoreactivity were detected in the neuropil, as considered typical of CJD 39, 59. In further cases, in addition to these small tau positive neuritic profiles, neuronal and glial tau pathology was also seen. In cases of group II (*n* = 30; 40%; 13 men; age: 72.6 ± 1.0) the distribution of neurofibrillary tau pathology in the subregions of the medial temporal lobe (MTL) was compatible with Braak stages 5. Group II comprised ten cases showing Braak stage I, eleven cases stage II, seven cases stage III and single cases with stages IV and VI. Careful inspection and evaluation of the subregional distribution of tau pathology indicated deviations from the original description of Braak stages in further 22 cases; therefore we distinguished these as a separate group (group III; *n* = 22; 29.3%; 8 men; age: 70.4 ± 1.7). Following the diagnostic strategy of the BrainNet protocol 1, in 19 cases the tau pathology did not expand beyond the border of the sulcus collateralis (Braak stage < III) and in three cases it was seen also in the inferior temporal cortex compatible with stage III. Finally, in group IV (*n* = 11; 14.7%; 9 men; age: 75.6 ± 1.0) further tau pathologies compatible with primary tauopathies or ARTAG were observed with various degree of neurofibrillary degeneration (four cases Braak stage II and seven stage III). The representation of molecular subtypes among these groups of tau pathologies (Figure 2A) and the representation of different tau pathologies in each molecular subtype (Figure 2B) was not significant; only VV‐2 cases showed a tendency to associate with type III tau pathology (Chi^2^ test, *P* = 0.07).

Age at death was significantly different between groups (K‐W test; *P* = 0.001; group I: 65.0 ± 1.4; II: 72.6 ± 1.0; III: 70.4 ± 1.7; IV: 75.6 ± 1.0), in particular between groups I and II (M‐W test; *P* = 0.003) and between groups I and IV (M‐W test; *P* = 0.001). Duration of illness (months) was longest for group IV (10.6 ± 4.4); however, this was not significantly longer that in other groups (K‐W test; *P* = 0.11; group I: 9.2 ± 1.6; II: 6.5 ± 1.2; III: 5.0 ± 0.8). Age and duration was significantly different between molecular types of sCJD (K‐W test; *P* < 0.01 for both). Therefore, we compared age and duration in MM/MV type 1 cases, which showed similar results (K‐W test; *P* = 0.03 for age and *P* = 0.6 for duration). In the total cohort, age correlated inversely with the duration of illness (Spearman's rank correlation coefficient rho, *R* = −0.3, *P* < 0.001) and positively with the Braak stages (Spearman's rank correlation coefficient rho, *R* = 0.6, *P* = 0.007). Duration of illness did not show correlation with Braak stages. Finally, in group III the total load of neuritic, but not neuronal cytoplasmic, tau pathology (sum of the scores for different subregions) showed correlation with age (Spearman's rank correlation coefficient rho, *R* = 0.48, *P* = 0.03) and inverse correlation with the duration of illness (Spearman's rank correlation coefficient rho, *R* = −0.43, *P* = 0.053). In summary, duration of illness and age at death was strongly influenced by the molecular subtype of CJD, exemplified by group I cases where 7 out of 12 cases were rare molecular subtypes (VV1, VV2, MV2, MM2 cortical) in younger patients with longer duration of illness. On the other hand, cases with the longest duration of illness were in group IV (cases 10 and 11 in Table 1; 29 and 48 months, respectively).

**Table 1:** Overview of clinicopathological and cerebrospinal fluid examination of sporadic and genetic Creutzfeldt‐Jakob disease (CJD) cases with widespread tau pathologies. Bold value indicates elevated CSF pTau. Abberviations: m = male; f = female; MM = methionine/methionine; VV = valine/valine; BB = Braak and Braak stage of neurofibrillary degeneration; AGD = argyrophilc grain disease; PART = primary age‐related tauopathy; PSP = progressive supranuclear palsy; AD = Alzheimer disease; ARTAG = ageing‐related tau astrogliopathy; WM = white matter; HS = hippocampal sclerosis; TDP = TAR‐DNA binding protein 43 kDa; INIBD = intranuclear inclusion body disease; B4 = Lewy–pathology Braak stage 4; CAA = cerebral amyloid angiopathy; na = not analyzed.

No.	Age	Sex	CJD	BB	Tauopathy	Remark	CSF t‐Tau (pg/mL)	CSF pTau (pg/mL)
1	77	m	MM2	3	AGD		1265	**74**
2	80	m	MM1	2	AGD		na	na
3	78	f	MV2	2	AGD	Lewy body disease B4	1200	**62**
4	79	f	MM1	3	AGD		2262	**81**
5	72	m	MM1	3	AGD + PSP		2294	56
6	75	m	MM1	3	AGD	Kuru plaques in WM + INIBD	2262	52
7	71	m	VV2	3	ARTAG + AD	Aβ plaques	1200	52
8	77	m	MM1	2	ARTAG + PART		2262	**74**
9	79	m	MM1	3	ARTAG + AD	HS + Aβ plaques	na	na
10	71	m	MM1+2	3	ARTAG +AD	HS + TDP + CAA + Aβ plaques	na	na
11	73	m	MM1	2	ARTAG + PSP	Lewy body disease B4	2262	51
12	76	m	V203I MM1	2	ARTAG + PART		2359	48
13	71	f	V203I MM1	3	ARTAG + PART		na	na

Aβ plaques were not detected in cases of group I. In group II a single case was compatible with Thal phase 1, eight showed Aβ deposits in the neocortex and additionally in the hippocampus (Thal 2) and a single case showed widespread deposition (Thal 5) 66. Twenty cases were compatible with definitive PART (66.6%). In group III three cases showed Thal phase 2 and four cases Thal phase 1. Fifteen cases were classified as definitive PART (68.1%). In group IV only three cases showed Aβ plaques. In the total cohort, deposition of Aβ correlated with age (*R* = 0.38, *P* = 0.001). Mild degree of cerebral amyloid angiopathy [CAA type 2, stage 1 64, 65] was detected in 10 (13.3%; group I: 2/12; group II: 5/30; group III: 1/22; group IV: 2/11).

### Distribution of hippocampal tau pathology defines a subset of sCJD cases

Based on the original description of tau pathology in the hippocampus in Braak stages, the dentate gyrus and CA4 subregions are presumed to be involved only in later Braak stages (mostly V and VI), furthermore, the entorhinal cortex precedes the involvement of the subiculum and also shows a higher load of tau pathology 50. Semiquantitative analysis of neuronal cytoplasmic and neuritic tau immunoreactivity in group III cases showed however different patterns based on the tau load: (i) In 15 cases the subiculum was equal or higher to that in the entorhinal cortex; (ii) in 10 cases the dentate gyrus tau‐load was equal or higher to that in the entorhinal cortex; and (iii) in 14 cases the CA4 subregion was equal or higher to that in the entorhinal cortex. In eight cases we observed overlapping patterns. Importantly, seven cases showed lack of tau pathology in the entorhinal cortex, therefore, we performed additional comparisons (EC+, *n* = 15; EC−, *n* = 7).

**Figure 3:**
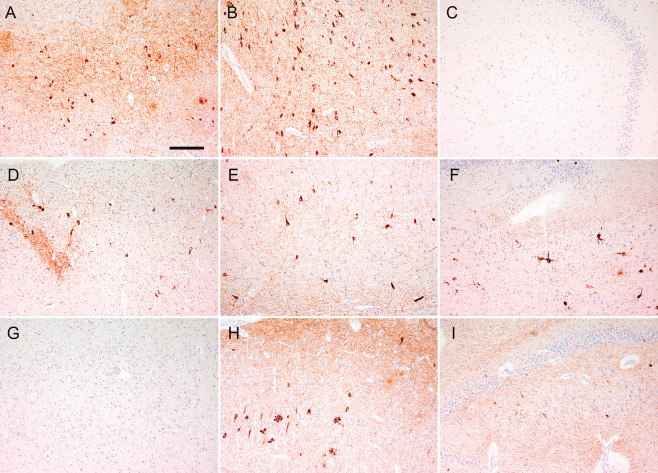
Immunohistochemistry for phospho‐tau (AT8) in the entorhinal cortex **(A, D, G)**, CA1 subregion of the hippocampus **(B, E, H)**, CA4 and dentate gyrus **(C, F, I)** in a representative case from group II (typical Braak distribution of tau pathology **(A–C)** and group III (atypical distribution of tau pathology) including entorhinal cortex (EC) positive **(D–F)** and EC negative **(G–I)** subtypes. Bar in A represents 120 μm for all images.

Comparison of the subregional neuronal cytoplasmic and neuritic tau immunoreactivity scores showed significant differences between group II (ie, pooled cases of Braak stage ≤ III) and group III. For the neuritic tau immunoreactivity scores K‐W test showed significant (*P* < 0.05) values for all subregions examined except CA3 (non‐hilar part). For the neuronal tau immunoreactivity scores only the subiculum, entorhinal cortex and dentate gyrus was significantly different. For group comparisons M‐W test showed significant differences for several subregions (Figure 2C,D). Based on the Braak staging concept that the entorhinal cortex is involved early, we next aimed to highlight the differences by the subtraction of the scores given for the entorhinal cortex and that given for the subiculum, dentate gyrus and CA4 subregion. This revealed highly significant (K‐W test, *P* < 0.001) differences for the neuritic and neuronal tau pathology scores between groups II and III (Figures 2E,F and 3A–I).

### Description of sCJD cases with widespread tau pathology

**Figure 4:**
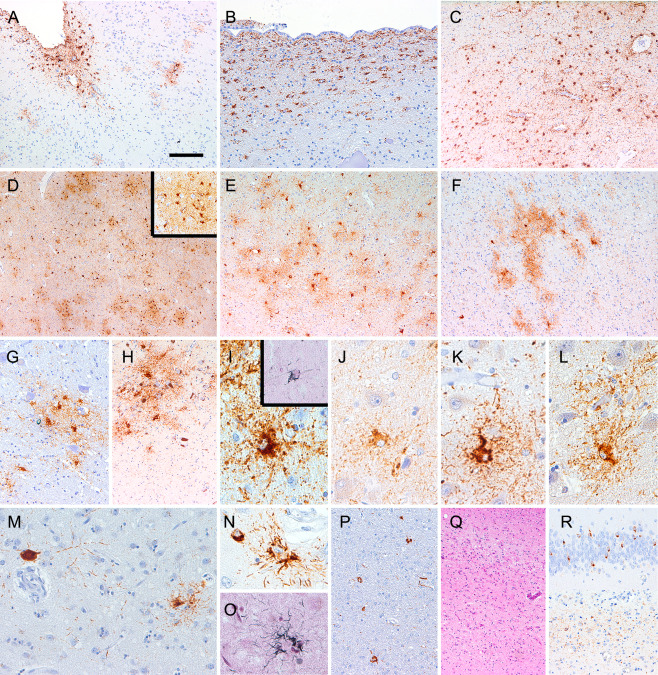
Immunohistochemistry for phospho‐tau (AT8) in representative sporadic CJD cases. Ageing‐related tau astrogliopathy (ARTAG) subpial (**A**; frontal cortex), subependymal (**B**; lateral ventricle), white matter and perivascular (**C**; amygdala) type. Gray matter type of ARTAG in the amygdala (**D**; enlarged inset with AT100 immunostaining), superior temporal gyrus **(E)**, nucleus accumbens **(F)**, nucleus hypoglossus **(G)**, substantia nigra **(H)**. Granular‐fuzzy astrocyte immunosatined by AT8 (**I**; upper inset shows Gallyas silver staining), AT270 **(J)**, AT180 **(K)** and AT100 **(L)**. Immunostaining for AT8 in the putamen (**M**; case 11) reveals a globose neurofibrillary tangle (left) and a tufted astrocyte (right), which is immunoreactive for 4R tau isoform **(N)** and Gallyas positive **(O)**. The same case shows oligodendroglial coiled bodies (**P**; frontal white matter). Hippocampal sclerosis (**Q**; CA1 subregion of hippocampus, case 10) and phospho‐TDP‐43 immunoreactive inclusions in the hippocampus **(R)**. Bar in A represents 120 μm for A, C, E, F, G, H; 40 μm for B, M, P, Q, R; 20 μm for I‐L, N, O; and 180 μm for D.

**Figure 5:**
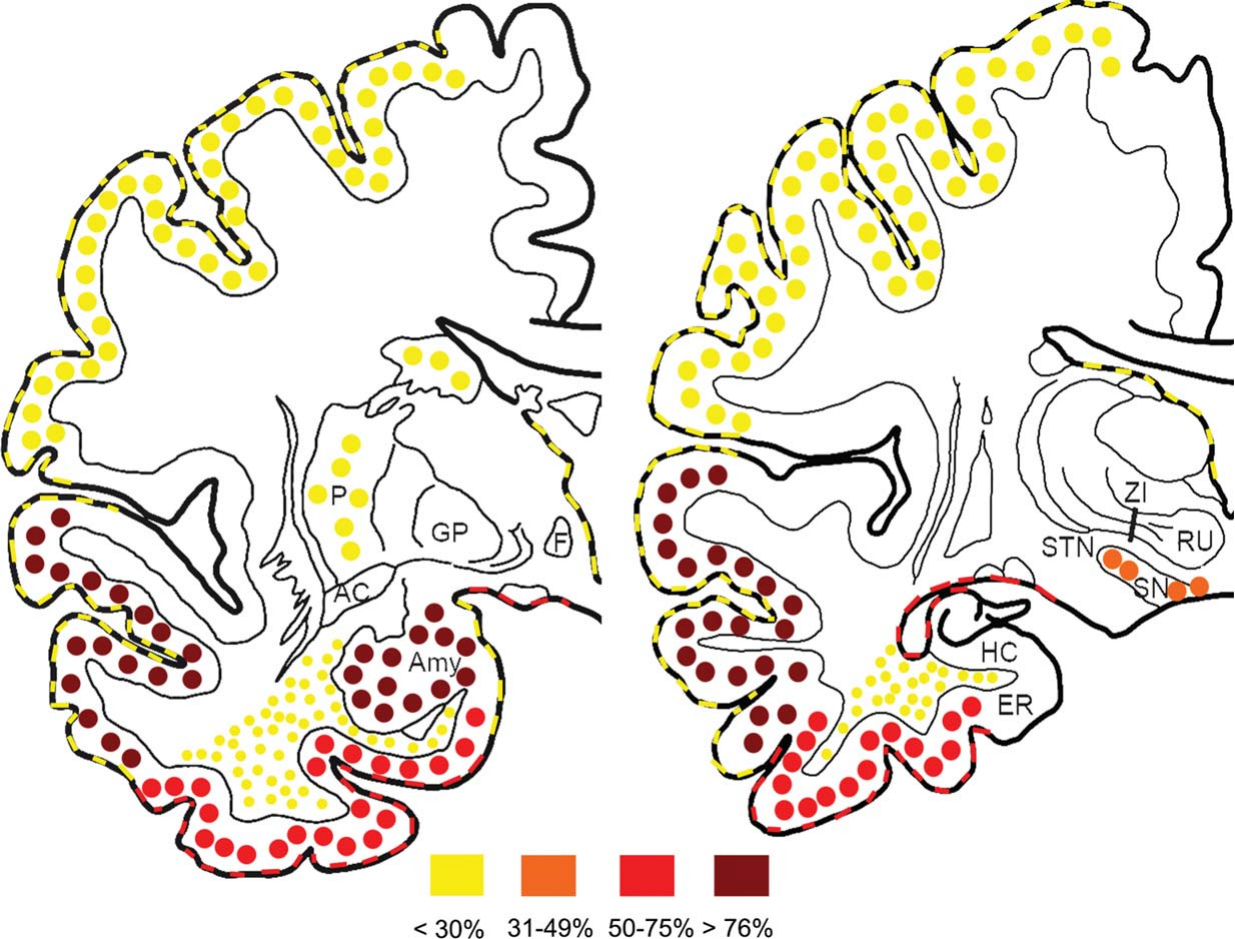
Heat mapping of subpial, subependymal, gray and white matter ageing‐related tau astrogliopathy (ARTAG). Color codes indicate the percentage of cases with ARTAG pathology showing these subtypes. Lines indicate subpial or subependymal, large dots indicate gray matter and small dots white matter ARTAG. Details on further anatomical areas and subregional distribution are summarized in Table 2.

Eleven cases showed more widespread tau pathology (Table 1). In eight cases CSF examination was performed, which revealed pathological elevation of total tau levels in all and increased pTau levels in four (Table 1). Six out of eleven showed changes compatible with AGD, including one with subcortical neurofibrillary tangles as in early forms of PSP. Five cases showed prominent astroglial tau pathology, including granular/fuzzy astrocytes (GFA) in gray matter regions, subpial, subependymal, perivascular and white matter thorn‐shaped astrocytes (TSA) compatible with ARTAG type pathology (Table 2 and Figures 4 and 5). Gallyas silver staining labeled a subset of the TSAs but not the GFAs. Immunostaining for further pTau antibodies showed similar patterns as AT8, except for AT270 (pT181), which revealed much less immunoreactivity, mainly in neurons. One of these had subcortical NFTs and argyrophilic tufted astrocytes in the cortex and striatum compatible with concomitant PSP type pathology (Table 2 and Figure 4). One case showed additional features of intranuclear inclusion body disease 4 and two Lewy bodies (Braak stage 4) 6. The distribution of spongiform change and the morphology of PrP deposition were not distinct when compared to CJD cases without widespread tau pathology.

**Table 2:** Distribution of tau immunoreactivities in sporadic and genetic Creutzfeldt‐Jakob disease (CJD) cases with ageing‐related tau astrogliopathy (ARTAG) type of tau pathologies. Case numbers refer to cases listed in Table 1. Abbreviations: Amyg = amygdala; BF = basal forebrain; BGG = basal ganglia; E = entorhinal cortex; FR = frontal; iTE = inferior temporal gyrus; infHorn = inferior horn of lateral ventricle; Hipp = hippocampus; LC = locus coeruleus; LV = lateral ventricle; MO = medulla oblongata; MTL = medial temporal lobe; Occ = occipital; PB = pons base; SN = substantia nigra; sTE = superior temporal gyrus; Subthal = subthalamic nucleus; Tegm = tegmentum of mesencephalon; Thal = thalamus. − indicates no; + indicates yes.

	Case	No. 7		No. 8		No. 9		No. 10		No. 11		No. 12 + 13	
	Region	Present	Region	Present	Region	Present	Region	Present	Region	Present	Region	Present	Region
ARTAG	MTL	−		−		+	iTE	+	iTE	−		+	Amyg/iTE
Subpial	Lobar	−		+	FR	−		+	FR	−		+	sTE
	Subcortical	−		+	BF	+	BF	+	BF	−		+	BGG
	Brainstem	−		−		−		−		−		+	Mesencephalon
ARTAG	MTL	−		+	LV/infHorn	−		+	LV/infHorn	+	LV/infHorn	+	LV/infHorn
Subependymal	Lobar	−		−		−		−		−		−	
	Subcortical	−		+	LV	−		−		−		+	3rd V
	Brainstem	−		−		−		−		−		−	
ARTAG	MTL	+	Amyg/iTE/E	+	Amyg	+	Amyg/iTE	+	Amyg	+	Amyg	+	Amyg/iTE
Gray matter	Lobar	+	sTE	+	FR+ sTE	+	sTE	+	FR/sTE	+	sTE	+	sTE/Occ
	Subcortical	−		−		−		+	BGG/BF	−		+	BGG/Thal
	Brainstem	−		+	SN/Tegm	+	SN/Tegm/MO	+	SN/Tegm	−		+	SN
ARTAG	MTL	−		−		+	Amyg	+	Amyg	−		+	Hipp
White matter	Lobar	−		−		+	Occ	−		−		+	Occ
	Subcortical	−		−		−		−		−		−	
	Brainstem	−		−		−		−		−		−	MO
ARTAG	MTL	+	Amyg	−		+	Amyg	−		−		+	Amyg
Perivascular	Lobar	−		−		−		+	FR	−		−	
	Subcortical	−		+	BGG	−		−		−		+	BGG
	Brainstem	−		−		−		−		−		−	
Tufted	MTL	−		−		−		−		−		+	Amyg
astrocytes	Lobar	−		−		−		−		+	FR/sTE	−	
	Subcortical	−		−		−		−		+	BGG	+	BGG/Thal
	Brainstem	−		−		−		−		−		−	
Coiled bodies	MTL	−		−		+	Amyg	−		−		+	Amyg
	Lobar	−		−		−		−		+	FR	−	
	Subcortical	−		−		−		−		+	BGG	−	
	Brainstem	−		−		−		−		−		−	
Grains	MTL	−		−		+	Amyg	−		+	Amyg	−	
	Lobar	−		−		−		−		−		−	
	Subcortical	−		−		−		−		−		−	
	Brainstem	−		−		−		−		−		−	
Neuronal	MTL	+	Amyg/Hipp	+	Amyg/Hipp	+	Amyg	+	Amyg/Hipp	+	Amyg/Hipp	+	Amyg/Hipp
	Lobar	+	sTE	−		+	sTe	+	sTE	+	FR	−	
	Subcortical	−		−		+	BGG	+	BGG	+	BGG	+	BGG, Thal/Subthal
	Brainstem	−		−		−	−	−		+	SN/Tegm/LC/PB	+	SN

### Description of V203I genetic CJD cases

**Figure 6:**
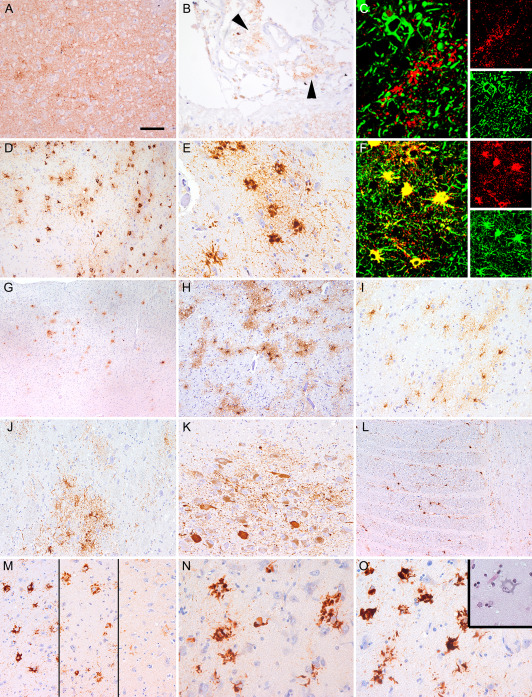
Neuropathological observations in genetic CJD (V203I). Immunostaining for PrP reveals diffuse synaptic deposits in the frontal cortex **(A)** but also in the leptomeninges (**B**; arrowheads). GFAP immunoreactive astrocytes (green) do not co‐localize with PrP deposits (red) **(C)**. Immunostaining for AT8 reveals gray matter type of ageing‐related tau astrogliopathy (ARTAG) in the amygdala **(D–F)**, frontal **(G)** and temporal **(H)** cortex, accumbens nucleus **(I)**, medial thalamus **(J)**, locus coeruleus (**K**; here together with neuronal tau pathology), and medial lemniscus in the medulla oblongata **(L)**. The AT8 (red) immunoreactive granular‐fuzzy astrocytes co‐localize with GFAP (green) (F). Astrocytic tau pathology is strongly visible using antibody AT180 (**M**; left), AT100 (M; middle) and weakly with AT270 (M; right). Astrocytes with thorny morphology immunostain for 4R tau isoform **(N)** and for p62 **(O)** and weakly for Gallyas (O; right upper inset). Bar in A represents 120 μm for A, B, D, H, L; 180 μm for G; 40 μm for I, J, K, M; and 20 μm for C, E, F, N, O.

During the collection of cases for this study we observed prominent astroglial tau pathology in an Austrian case with the *PRNP* mutation V203I M129M. To evaluate whether this is seen only in a single case, we included two further unpublished cases from France (Lyon) with the same mutation. Duration of illness was 2, 4 and 7 months, respectively. Clinical symptoms included progressive dementia, myoclonus, pyramidal/extrapyramidal symptoms in all three, cerebellar ataxia and akinetic mutism in two, visual impairment in one, falls and hallucination in another one (see *Supporting Information*). All three cases showed moderate to prominent spongiform degeneration, neuronal loss and gliosis in the entire cortex, basal ganglia, thalamus and cerebellar cortex. Immunostaining for disease‐associated PrP revealed a uniform pattern, consisting of diffuse synaptic, perineuronal and somatosynaptic depositions, including the leptomeninges (Figure 6A,B). PrP did not co‐localize with GFAP (Figure 6C). Neocortical Aβ deposits were detected in case 1. Immunohistochemistry for pTau (AT8) revealed in two cases a complex tauopathy, with predominantly gray matter type of ARTAG pathology (Table 2 and Figure 5). Tau pathology was characterized by clusters of GFAs, showing co‐localization of pTau and GFAP (Figure 6D–K). Thorny astrocytes were observed in the white matter including the medulla oblongata (Figure 6L). Immunostaining for further pTau antibodies showed similar patterns as AT8, except for AT270, which revealed much less immunoreactivity (Figure 6M). In the amygdala and hippocampus the pTau‐ and 4R tau isoform (Figure 6N) immunoreactive astrocytes showed bulky cytoplasm, and thorny and tufted‐like appearance. These were immunoreactive for p62 and partly also argyrophilic (Gallyas). Moreover, NFTs, pretangles, neuropil threads and oligodendroglial coiled bodies were also visible. Except for the neurofibrillary tangles (3R + 4R), tau pathology was predominated by the 4R isoform and involved the hippocampus, amygdala, frontal and temporal cortex, and to a lesser extent subcortical regions including basal ganglia and thalamus. Despite the high amount of tau pathology, in the CSF only total tau levels were increased but not the pTau (T181) level (which is represented by the AT270 immunohistochemistry) (Table 1). Finally, the third case showed only moderate amounts of small neuritic profiles.

## Discussion

The present study highlights different patterns of tau pathology in sCJD and in cases with the V203I *PRNP* mutation. The spectrum of pTau immunoreactivities observed here comprise (i) neuritic profiles; (ii) neuronal tau pathology restricted to the medial temporal lobe with or without deviation from the classical distribution described by Braak and Braak; (iii) features of primary tauopathies; and (iv) mostly the gray matter type of ARTAG. There are brains, which show combinations of these morphologies. In the present paper we evaluated sCJD and selected genetic CJD cases and did not include cases with prominent PrP amyloid deposits such as GSS where the pathogenesis of tau pathology is associated with prominent multicentric amyloid plaques 14, 15, 16, 17, 18, 19, 20. Recently, we published a large cohort of E200K mutation cases and described combined pathologies including mostly neuronal tau pathologies 46. Therefore, in the present study we focused on sCJD. The reason why we included V203I mutation genetic CJD is the similarity of tau pathology seen in a subset of sCJD cases (gray matter ARTAG).

Neuritic profiles are the most frequent type of tau immunoreactivity in CJD brains. A recent study 59, which evaluated only the frontal cortex and the cerebellum suggested strong correlation with the density of PrP immunodeposition but not with the duration of illness. The authors proposed that PrP load might be the triggering factor for tau phosphorylation 59. On the other hand, it might reflect only a secondary process, namely the degeneration of neurons where the damaged neurites accumulate variable but mostly small amounts of phosphorylated tau. Tau immunoreactivity was described also surrounding kuru type plaques in the rare MV type 2 sCJD subtype as seen also in kuru affected brain 62. Interestingly, pTau‐immunoreactive neuritic profiles around PrP amyloid deposits are seen in the acquired variant CJD as well 21. Contrasting AD‐related amyloid‐β (Aβ deposition, no paired helical filaments can be observed in dystrophic neurites on electron microscopy 62. In contrast, in iatrogenic CJD related to dura mater transplantation, mature Aβ plaques do not harbor a tau‐immunoreactive neuritic corona 42, 57. Finally, the multicentric PrP‐amyloid plaques in GSS are frequently surrounded by tau‐immunoreactive dystrophic neurites together with variable degree and extent of neurofibrillary degeneration 14, 15, 16, 17, 18, 19, 20.

Neurofibrillary degeneration is a constant feature of the aging brain, which follows hierarchical distribution described in the frame of AD‐related pathologies. In the medial temporal lobe, the entorhinal cortex is one of the earliest anatomical locations showing this pathology 5. In the cornu ammonis of the hippocampus the CA1 subregion is the most affected, while the CA4 subregion together with the granular layer of the dentate gyrus is involved only in later stages (ie, V–VI) 5, 50. In NFT dementia cases, contrasting AD, NFTs predominate in all hippocampal subregions 34 but without significant amounts of Aβ deposition in cortical areas as in AD. Indeed, NFT dementia is included in the recent concept of PART implicating that in early stages of neurofibrillary degeneration it cannot be decided whether a case will develop AD with Aβ or remains in the NFT‐only state 8, 31. NFT pathology is frequently seen in GSS and particularly in mutations with PrP‐CAA. The tau immunoreactivity profile and ultrastructure is very similar if not identical to AD 16, 19, 22. Tau pathology in GSS seems to relate to PrP deposition as seen in other amyloidoses 25. This supports the concept that abnormal tau phosphorylation accompanies cerebral amyloid deposition regardless of the chemical composition of the amyloid. In some mutations with GSS the distribution of NFT pathology deviates from that seen in AD and involves more cortical areas or even brainstem, thalamus and amygdala 52, 71. There is a paucity of systematic evaluation of tau pathology in sCJD. Co‐existence of AD and CJD is seen in larger series 23, 36, 67, 68. A comprehensive study following the CERAD (Consortium to establish Registry for AD) criteria and applying silver staining 51, indicated that definite and probable AD constituted 10.9% of sCJD cases, somewhat lower as in the control group (19%) 24. Further studies theorized that disease‐associated PrP induces Aβ deposition, at least in a subset of cases (17% in that study) 67. The present study, however, shows that in sCJD tau pathology is more frequent than Aβ pathology. Our cohort also included elderly cases without Aβ deposition but with tau pathology. This is significantly different from iatrogenic CJD, where the frequency 11, the distribution and morphology of Aβ pathology is clearly distinct and completely lacks neurofibrillary degeneration 42. In genetic CJD with the E200K mutation, the distribution of hippocampal neurofibrillary pathology shows distinct patterns 46. A recent study indicated that non‐AD type tau pathology can be frequently seen in sCJD 23. The present study revealed different hippocampal tau pathology in sCJD. The distinct involvement of the entorhinal cortex together with the increased tau deposition in the dentate gyrus and CA4 subregion [which is considered to contain pyramidal cells with similar cytoarchitecture and connectivity as those in CA3 but enclosed by the dentate gyrus 27] in a significant subset of sCJD cases indicates a deviation from the distribution seen in typical AD cases. Indeed, in some aspects this is more reminiscent of other tauopathies like Pick's disease or even AGD, PSP or CBD 50. The entorhinal cortex projects mostly to the CA3 subregion and dentate gyrus, but other layers have connections to the CA1 and subiculum 27. Accordingly, a distinct spreading pattern of tau pathology within intrinsic loops of the hippocampus might be considered in a subset of sCJD cases. Alternatively, local initiation of tau hyperphosphorylation, which is not strictly related to accumulation of disease‐associated PrP, could be discussed. Importantly, deviations from the AD‐type involvement of NFT pathology in hippocampal subregions have been reported for Lewy body disorders 26, 29.

Astrocytic tau pathologies have been underappreciated in CJD. A systematic study evaluating a large cohort of E200K *PRNP* mutation genetic CJD emphasized that widespread tau pathology characterizes a subset of cases 46. Co‐localization studies indicated that these are related to neurons and neurites but not to astrocytes. The present study is the first to reveal prominent astrocytic tau pathology in sCJD and also in V203I *PRNP* mutation genetic CJD. Some of these cells are reminiscent of tufted astrocytes, which together with subcortical NFTs, are compatible with PSP 34. However, there are further ones, including granular‐fuzzy astrocytes and thorn‐shaped astrocytes often appearing in clusters in the gray matter. Additionally, thorny astrocytes in subpial, subependymal, white matter and perivascular locations have been also seen. These immunoreactivities are compatible with the recently defined ARTAG 40. The widespread distribution of gray matter type ARTAG in the cortical and subcortical limbic areas and brainstem is similar to that described in a cohort of elderly demented individuals 43, 45. The immunohistochemical tau signature and the inconsistent Gallyas silver positivity distinguish these astrogliopathies from the astrocytic plaques of CBD and tufted astrocytes of PSP 10, 43, 45. The anatomical areas (ie, amygdala, hippocampus, accumbens nucleus, substantia nigra) affected by this tau pathology deviates from the regions typically vulnerable in sCJD. This suggests an independent process, namely that CJD appeared in a brain with already ARTAG or PSP pathology. Accordingly, these are not the reactive astrocytes seen extensively in CJD brains. In addition, our study did not find any effect of the duration of illness on the presence of tau pathology. Interestingly, similar complex astrocytic tau pathologies overlapping with ARTAG morphologies have been reported in *TARDB* mutation 13 and in familial disorders without a specific gene mutation 2, 9. Of note however, CJD is a very rare disease and the chance of concomitant affection by other rare disorders such as PSP is very low. Nevertheless, similar rare constellations are known for PSP and MSA 43, 63, for CJD and INIBD 4, 23 and for CJD with MSA 23. Furthermore, hippocampal sclerosis with TDP‐43 pathology has not yet been described in CJD, which usually lacks TDP‐43 immunoreactive inclusions 23, 28. While these constellations raise the possibility that these are not by‐chance events, the low number of these tauopathies argues against a common effect of prion disease‐related pathogenesis. It is important to note that prion disease may appear in long duration neurodegenerative conditions and present as an acceleration of the clinical course. Because some of the cases with these predominantly age‐related pathologies are below 75 years of age, one could speculate that the ageing process is accelerated in some CJD brains.

Genetic CJD associated with the V203I mutation and homozygosity for Met at codon 129 was first described only with clinical data 55. Further cases were reported from China 61 and Japan, including a homozygous case for the mutation 33 and neuropathological description of a case 32. Here we describe three new cases of V203I‐129M, showing classical neuropathological features of CJD. Moreover, two of the patients showed a concomitant unusual 4R tauopathy with peculiar astrogliopathy. Only one showed clinical symptoms, such as falls and hallucinations, that were not characteristic for CJD.

Examination of total tau and pTau protein levels in the cerebrospinal fluid is an established method for AD diagnostics. In sCJD, protein 14‐3‐3 is the best performing surrogate laboratory marker. However, as confirmed here, total tau protein presents comparable levels of sensitivity and specificity 58. In contrast, evaluation of pTau in CJD is less helpful, and as we show it does not reliably reflect the tau pathology in CJD brains. This might be caused by the fact that the pTau epitope examined in the routinely used assays labels the least of tau pathological alterations in the post‐mortem examination. This observation has important implications for the application of this epitope for dementia diagnostics. In particular, pTau epitopes vary considerably even during the same disease process 35. Thus, depending on the tau pathological process (neuronal or glial predominant or mixed forms) 34, CSF pTau examination with a single epitope marker might not reflect the whole spectrum of alterations. Tau in CSF occurs as a series of fragments; discrimination of AD from controls is dependent on the subset of tau species measured 49. There are studies examining further epitopes (eg, T231) 3, 7, which we evaluated here, however, there is a relative paucity on data on their reliability and reproducibility. Indeed, CSF and brain pTau patterns have not been compared systematically. More pTau epitopes should be measured in the CSF to reflect better the spectrum of tau pathologies in the brain.

There is a paucity of observations on the interaction of tau protein and PrP. Gene knockout of tau did not contribute to the pathogenesis of prion disease in mice 48. No evidence was found for an association between *MAPT* gene variations and sCJD, and only some weak evidence for an association with vCJD 60. Our early study on the physiological cellular PrP and pTau in tauopathies did not reveal co‐localization in tau‐positive inclusions 47. On the other hand, PrP 106–126 peptides are thought to induce glycogen synthase kinase 3β (GSK3β) mediated tau phosphorylation 56. A study on scrapie‐infected hamsters revealed that changes of pTau are more prominent as in controls 69. According to *in vitro* observations 70 binding activities of a PrP‐Tau complex might differ between *PRNP* mutations, explaining the variability of tau pathologies. Most likely, the rapid pathogenesis of sCJD does not allow complex interactions leading to significant amounts of tau pathologies. While intraneuronal processing of PrP involves similar systems as in other neurodegenerative conditions 41, in the case of mutated PrP the longer and constant process 44 might lead more efficiently to concomitant proteinopathies.

The spectrum of tau pathologies in prion diseases can be summarized as follows 39: (i) small neuritic profiles; (ii) dystrophic neurites around multicentric PrP amyloid plaques in GSS and variant CJD; neurofibrillary degeneration, (iii) following or (iv) deviating from the distribution described by Braak and Braak 5; (v) Widespread neuronal and neuritic tau pathology (ie, E200K genetic CJD or FFI, and GSS or PrP‐cerebral amyloid angiopathy) including neocortical and subcortical areas; (vi) features of primary tauopathies (eg, AGD, PSP); and (vii) features of ARTAG, mostly gray matter type (eg, genetic CJD and sCJD).

## Conclusions

The frequency of neuronal and glial tau pathologies is not unusually high in CJD and does not precisely relate to PrP deposition. PART‐type pathology 8 is more frequent than AD with Aβ plaques. The pattern of hippocampal tau pathology often deviates from the stages by Braak and Braak 5. This has implications for understanding the hierarchical spreading of tau pathology and raises the possibility that there are several modes of development of tau pathology in the entorhinal cortex and hippocampal regions 50. Our study supports the observations that genetic CJD might associate with a wide spectrum of concomitant pathologies 46. Considerable variations between cases suggest further genetic/epigenetic influences. Finally, currently applied examination of CSF pTau (T181) level does not reliably reflect tau pathologies seen in CJD brains.

## Supporting information

Additional Supporting Information may be found in the online version of this article at the publisher's web‐site:

Summary of clinicopathological observations in three patients with V203I PRNP mutation. Abbreviations: n.d. = not done; BGG = basal ganglia; PSWC = periodic sharp wave complexes.
