# Supplementary material for: Tau pathology in Creutzfeldt‐Jakob disease revisited
Source: Brain Pathol. 2016 Aug 2;27(3):332–44. doi: 10.1111/bpa.12411 (PMC8028936; doi:10.1111/bpa.12411)
Supplement: Supplementary file 1 — Summary of clinicopathological observations in three patients with V203I PRNP mutation. Abbreviations: n.d. = not done; BGG = basal ganglia; PSWC = periodic sharp wave complexes. [file BPA-27-332-s001.pdf]

Online supplemental file 1: Summary of clinicopathological observations in three patients with V203I *PRNP* mutation. Abbreviations: n.d.: not done; BGG: basal ganglia; PSWC: periodic sharp

	Case 1	Case 2	Case 3
Age at death	76 years	71 years	69 years
Gender	man	woman	man
Disease duration	4 months	7 months	2 months
PRNP	V203I<br>M129M	V203I<br>M129M	V203I<br>M129M
Country of origin	Austria	France	France
Clinical symptoms			
Progressive dementia	+	+	+
Myoclonus	+	+	+
Pyramidal and extrapyramidal symptoms	+	+	+
Ataxia	+	-	+
Visual impairment	+	-	-
Falls, dizziness, hallucinations	-	+	-
Akinetic mutism	+	+	-
CSF 14-3-3	positive	positive	positive
MRI	n.d.	High signal in BGG and parietal cortex	n.d.
EEG	PSWC	PSWC	PSWC
Tau pathology	Complex	Complex	Neuritic profiles
